# Multidimensional imaging of liver injury repair in mice reveals fundamental role of the ductular reaction

**DOI:** 10.1038/s42003-020-1006-1

**Published:** 2020-06-05

**Authors:** Kenji Kamimoto, Yasuhiro Nakano, Kota Kaneko, Atsushi Miyajima, Tohru Itoh

**Affiliations:** 1 Laboratory of Stem Cell Therapy, Institute for Quantitative Biosciences, The University of Tokyo, 1-1-1 Yayoi, Bunkyo-ku, Tokyo 113-0032 Japan; 2 Present Address: Department of Developmental Biology, Washington University School of Medicine in St. Louis, St. Louis, MO 63110 USA; 3 Present Address: Department of Pathology, University of California San Diego, La Jolla, CA 92093 USA

**Keywords:** Adult stem cells, Bile ducts, Organogenesis, Regeneration

## Abstract

Upon severe and/or chronic liver injury, ectopic emergence and expansion of atypical biliary epithelial-like cells in the liver parenchyma, known as the ductular reaction, is typically induced and implicated in organ regeneration. Although this phenomenon has long been postulated to represent activation of facultative liver stem/progenitor cells that give rise to new hepatocytes, recent lineage-tracing analyses have challenged this notion, thereby leaving the pro-regenerative role of the ductular reaction enigmatic. Here, we show that the expanded and remodelled intrahepatic biliary epithelia in the ductular reaction constituted functional and complementary bile-excreting conduit systems in injured parenchyma where hepatocyte bile canalicular networks were lost. The canalicular collapse was an incipient defect commonly associated with hepatocyte injury irrespective of cholestatic statuses, and could sufficiently provoke the ductular reaction when artificially induced. We propose a unifying model for the induction of the ductular reaction, where compensatory biliary epithelial tissue remodeling ensures bile-excreting network homeostasis.

## Abstract (web summary)

Kenji Kamimoto et al. use multidimensional imaging technologies to study changes in the mouse biliary system following liver injury. They find an unexpected role of the ductular reaction – the process of ectopic expansion of biliary-like cells following liver injury – in restoring functional biliary structures in injured livers.

## Introduction

The liver epithelial tissue, consisting of hepatocytes and biliary epithelial cells (BECs), is generally quiescent in healthy conditions, yet manifests vigorous proliferative activities and plasticity in response to various types of insults, whereby constituting the remarkable regenerative potential of the liver^1,2^. Upon severe and/or chronic liver injury, ectopic emergence and expansion of atypical biliary epithelial-like cells in the liver parenchyma, known as the ductular reaction, is typically induced and implicated in organ regeneration^3–5^. The ductular reaction is histopathologically observed in a wide range of human liver disease conditions, and the degree of its induction has been suggested to correlate with the disease severity^6,7^. Studies using knockout mouse models defective in critical signaling pathways for induction of the ductular reaction have collectively shown that its suppression generally exacerbates liver injury and dysfunction and often affects organismal survival^8–10^, suggesting a pro-regenerative role of the ductular reaction in liver pathophysiology.

The ductular reaction has long been postulated to represent the activation of facultative liver stem/progenitor cells that reside in the biliary compartment and give rise to new hepatocytes^11,12^, so that the relationship between the ductular reaction and liver regeneration has been intensively studied with primary focus on cell lineages or cell supply^13,14^. In recent years, in vivo lineage-tracing analyses in mice have disclosed that hepatocyte renewal from the biliary epithelium is actually a rare event induced only under limited injury conditions^15–19^. Thus, induction of the ductular reaction upon liver injury does not inevitably lead to the stem/progenitor cell activation, thereby leaving its fundamental role and the mode of action in liver regeneration enigmatic.

In a landmark paper in 1957 describing the ductular reaction (or ‘ductular cell reaction’), Popper et al.^20^ already alluded that the sprouting and ramified ductular epithelia formed luminal structures that were connected to the bile duct. Later studies employing retrograde resin-casting and/or ink injection experiments via the extrahepatic common bile duct demonstrated that the expanded biliary branches possessed luminal structures that were physically connected to those of the pre-existing intrahepatic bile duct and ultimately to the extrahepatic biliary tract leading to the duodenum^21–24^. Three-dimensional (3D) tissue imaging analyses further revealed that the ductular reaction represents dynamic remodeling of the contiguous epithelial tissue structure of the biliary tree^22^. It remains to be clarified, however, whether newly formed biliary branches in the ductular reaction function as a practical biliary tract with anterograde bile flow emanating from hepatocytes, as well as how the nature of bile excretion could potentially be linked to the structural dynamics of the biliary epithelium.

Here, we address these issues by exploiting multidimensional imaging technologies to study structural and functional dynamics of the biliary system in mouse models of liver injury. Our intravital imaging system visualizes bile flow in the living mouse liver, which confirms that the expanded biliary epithelial tissue in the ductular reaction serves as functional bile-excreting ducts. Our imaging analyses also reveal that the collapse of the bile canalicular network in the liver parenchyma is induced at the early phase of liver pathology in both cholestatic and non-cholestatic liver injury conditions, and that the areas for the bile canalicular collapse and the biliary epithelial tissue expansion are statistically correlated. Based on these observations, we propose a unifying model for the role of the ductular reaction: it represents an adaptive and compensatory biliary epithelial tissue remodeling process to reconstruct functional bile-excreting network in place of the lost bile canaliculi in the injured liver. Finally, CRISPR/Cas9-based in vivo gene knockout experiment demonstrates that the loss of bile canalicular network induces biliary epithelial tissue expansion and remodeling, lending further support to our proposed model for the ductular reaction.

## Results

### Biliary channel network is collapsed upon liver injury

**Fig. 1:**
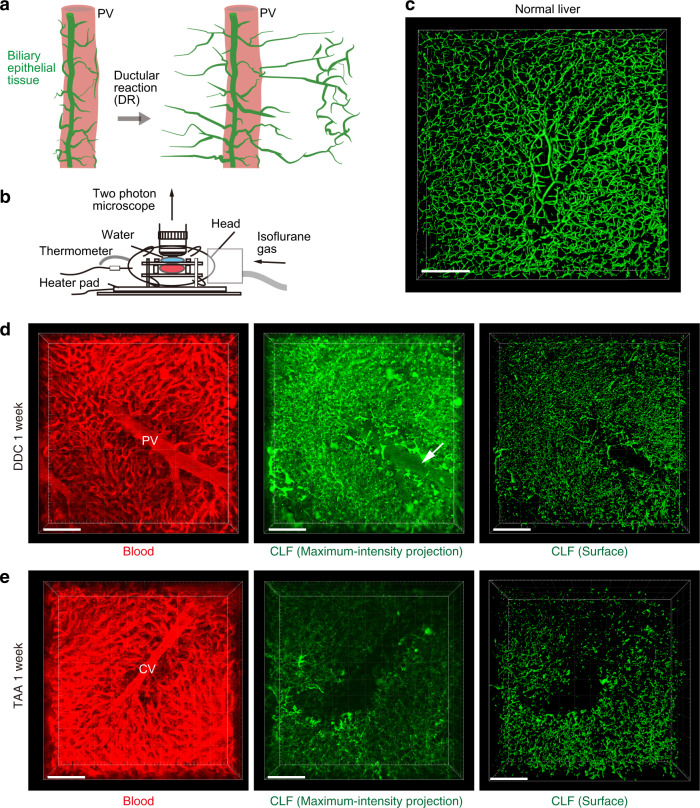
Observation of the bile-excreting channel structure in live mouse livers. **a** Schematic illustration of the ductular reaction. While biliary epithelial tissues (green) exist around the portal vein (PV, pink) in normal condition, they expand branches outward upon parenchymal injury. **b** Experimental setup for intravital imaging. Mice were anesthetized with isoflurane gas. The abdomen was incised to expose the liver, which was then attached to the developed liver-holding device. **c** Representative 3D image of the biliary channel structure under normal conditions, visualized with cholyl-lysyl-fluorescein (CLF) and captured with a two-photon microscope (*n* = 10 mice). Stacked images were used to reconstruct a 3D image using IMARIS software. The image is shown in surface mode, which highlights the surface of the object. **d**, **e** Representative images of blood flow and the biliary channel structure visualized with Texas red-conjugated dextran and CLF, respectively. Wild-type mice at 1 week of liver injury induced by DDC (**d**) or TAA (**e**) administration were subjected to intravital imaging (*n* = 8 mice for each). PV and CV denote blood vessels of the portal vein and the central vein, respectively. White arrow indicates bile accumulation in the portal vein in the DDC-injured liver. Scale bars, 100 μm.

To investigate the native and functional structure of biliary channels, where bile flows, we employed intravital imaging analyses in the mouse liver using two-photon microscopy (Fig. 1b). The liver was exposed and attached to an originally developed organ-holding device to minimize any liver movement caused by the heart rhythm and breathing and thus enable high-resolution imaging. We first visualized bile-excreting networks in healthy-mouse liver using a fluorescent-labeled bile analog, cholyl-lysyl-fluorescein (CLF)^25^. Bile is synthesized in hepatocytes and secreted into microchannels termed bile canaliculi, and the interconnected microchannels form a bile canalicular network, the ends of which directly connect with those of the bile duct. By using our experimental setting, the fine and ordered 3D network of functional biliary channels, composed of the bile canalicular network and the biliary tree, was clearly observed (Fig. 1c).

We then examined structures of the biliary channel under liver injury conditions, employing two representative types of liver disease models with different etiologies: the 3,5-diethoxycarbonyl-1,4-dihydrocollidine (DDC)-containing diet model and the thioacetamide (TAA) administration model. While both of these models have been used in common for the study of the ductular reaction and induce the biliary epithelial tissue remodeling and expansion over the duration of the injury (Supplementary Figs. 1 and 2), the resultant 3D structure of the tissue are quite diverged^22^. Of note, the induction of the ductular reaction is reversible at least until a certain period of time for injury, and the expanded biliary epithelial tissue can regress upon cessation of drug administration (Supplementary Fig. 3).

The DDC protocol is well established to induce cholestatic liver injury conditions in mice^26^, as manifested by massive elevation of serum cholestatic markers (Supplementary Fig. 4). Consistently, intravital observation revealed that the biliary channel structure was impaired broadly in the liver of mice fed DDC-containing diet (Fig. 1d). Overall bile accumulation was detected, including in the blood vessel, reminiscent of the cholestatic condition (Fig. 1d, white arrow in the central panel).

TAA administration represents a widely used protocol for the induction of experimental liver fibrosis^27,28^ and is considered a non-cholestatic model, with only a modest increase in serum cholestatic markers (Supplementary Fig. 4; Note that the *Y*-axis scales in the graphs are different between the DDC and TAA models). After 1 week of TAA administration, however, the biliary channel structure changed dramatically (Fig. 1e, middle and right panels) as compared with the normal condition (Fig. 1c). The collapse of the biliary channel was localized around the central vein (CV), consistent with the notion that TAA damages hepatocytes only around the CV^28^, and no severe accumulation of bile in the blood vessel was observed. Thus, by using the high-resolution intravital imaging system, cryptic failure of bile transportation was detected even in this non-cholestatic liver injury model.

### Ductular reaction induces expansion of bile-excreting ducts

**Fig. 2:**
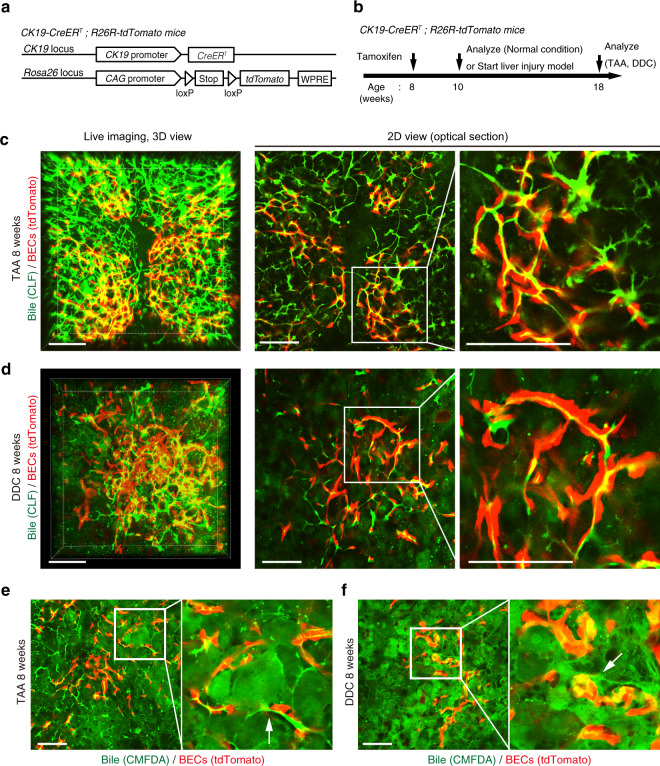
Expanded biliary structures in the ductular reaction function as bile-excreting channels. **a** CK19-CreERT;R26R-tdTomato mice were used to visualize biliary epithelial cells (BECs) with a red fluorescent protein. **b** Experimental design. BECs were labeled with tdTomato at 8 weeks of age and were then subjected to chronic liver injury (TAA or DDC). After 8 weeks of liver injury, the mice were analyzed by intravital imaging. Cholyl-lysyl-fluorescein (CLF)was injected immediately before intravital imaging. **c**, **d** Representative images of intravital observation of the biliary channel structure (CLF, green) and BECs (tdTomato, red) in the ductular reaction induced in the TAA (**c**) and DDC (**d**) models (*n* = 8 and 5 mice, respectively). The left panels show 3D reconstructed images, and center and right panels show pictures of a 2D optical section. Right panels are magnified views of the center images. Scale bars, 100 μm. **e**, **f** Representative images of intravital observation of chloromethyl fluorescein diacetate (CMFDA, green), which is metabolized in hepatocytes to form a fluorescent metabolite, and BECs (tdTomato, red) in the ductular reaction induced in the TAA (**e**) and DDC (**f**) models (*n* = 4 mice for each). Right panels are magnified views of the center images. Scale bars, 100 μm.

During week 1 of either of these models, the ductular reaction did not fully occur yet (Supplementary Figs. 1 and 2). The fact that defects in the biliary channel structures commonly existed under quite different liver injury conditions and preceded the ductular reaction induction led us to hypothesize that the failure of bile excretion in the early phase of chronic liver injury triggers a ductular reaction to recover the excretion by newly generating functional biliary epithelial tissue networks that complement the collapsed bile canalicular networks. To test this notion, the biliary channel structure and newly formed BECs were simultaneously visualized in situ by employing a transgenic mouse strain (CK19-CreERT; R26R-tdTomato) expressing a red fluorescent reporter protein in the BECs (Fig. 2a). Of note, the labeling efficiency in BECs was ~30–40% in this strain; thus, not all of the biliary trees could be visualized due to this experimental constraint. The mice were analyzed by intravital imaging at 8 weeks of DDC- or TAA-induced liver injury, when the ductular reaction was fully achieved (Fig. 2b). In both of these models, tdTomato^+^ cells overlapped with the signal for CLF (Fig. 2c, d), demonstrating that newly formed biliary trees have functional luminal structures that convey bile. Analysis using chloromethyl fluorescein diacetate, which is metabolized in hepatocytes to form a fluorescent metabolite that is eliminated via the bile canaliculi^29^, confirmed that the dye was secreted from hepatocytes into the luminal structure formed by BECs (Fig. 2e, f).

### BECs expand to compensate for the loss of bile canaliculi

**Fig. 3:**
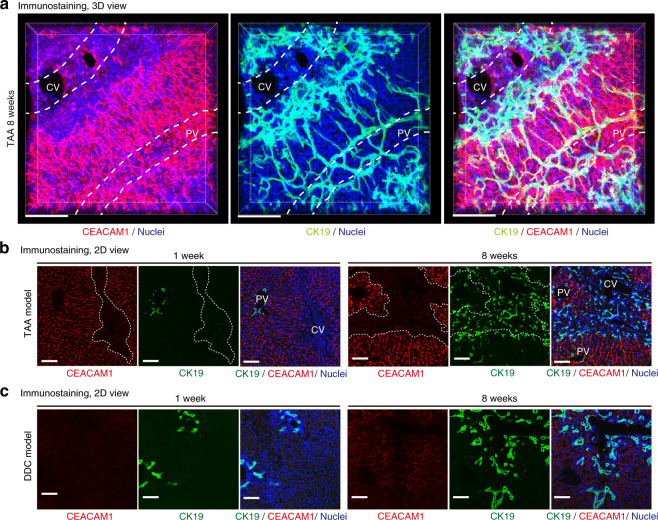
The extended biliary tree complements the lost bile canalicular networks upon parenchymal injury. **a** Representative image of 3D visualization of bile canaliculi (CEACAM1, red), bile ducts (CK19, green), and nuclei (Hoechst3342, blue) in the liver of the TAA injury model (*n* = 8 mice). Positions of blood vessels of the portal vein (PV) and the central vein (CV) are highlighted in white dotted lines. Scale bars, 100 μm. See also Supplementary Fig. 6 for images from different angles of view, showing the presence of blood vessel lumens. **b**, **c** Representative images of 2D immunostaining of injured mouse livers in the TAA (**b**) and DDC (**c**) models (*n* = 4 mice, respectively). In (**b**), collapse areas of bile canaliculi around the CV are highlighted with white dotted lines. Scale bars, 100 μm.

We then analyzed the spatial relationship between the biliary epithelial tissue and bile canaliculi by 3D co-immunostaining for corresponding markers. Under the normal and steady-state condition, the biliary epithelial tissue resides specifically around the portal vein (PV), forming a tree-like structure (Fig. 1a). Upon TAA-induced liver injury, the biliary tree initially extends branches toward the contralateral CV area where the injury occurs, and then expands therein to form network-like structures^22,30^, as exemplified by staining for the biliary marker CK19 or EpCAM and the peri-CV hepatocyte marker glutamine synthetase (Fig. 3a, Supplementary Figs. 5 and 6). Remarkably, a highly fragmented staining pattern for the canalicular marker CEACAM1 revealed that the bile canalicular structures were collapsed around the CV (Fig. 3a, b), consistent with the intravital observation of the biliary channel (Fig. 1e). This collapse was not due to the loss of hepatocytes upon injury, as many hepatocytes indeed existed in the collapsed area (Supplementary Fig. 7b). Thus, the hepatocytes in this area had lost their bile canalicular network structure. Importantly, the expanded area of the biliary tree and the area of collapsed bile canaliculi apparently overlapped (Fig. 3a, b). The collapse of bile canaliculi was also seen in DDC liver, although the collapse pattern was different from that in the TAA model (Fig. 3c). In both models, the collapse of bile canaliculi was induced much earlier than the expansion of the biliary epithelium. Moreover, in the recovery phase of these injury models upon withdrawal of the causative toxins, the regression of the ductular reaction coincided with the reconstitution of bile canaliculi (Supplementary Fig. 3). These data comprehensively support our hypothesis that the ductular reaction is induced to form new functional bile-excreting networks in the parenchymal area where bile canaliculi are collapsed by injury.

**Fig. 4:**
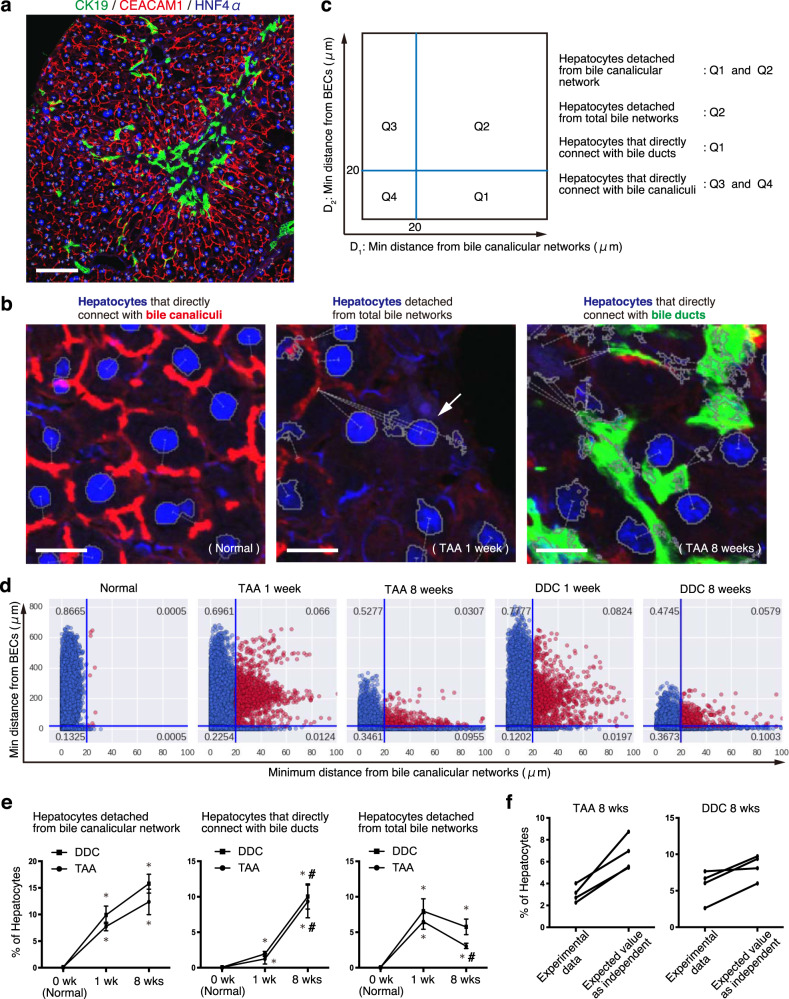
Hepatocytes detached from bile canalicular networks re-establish connection with the network of the bile duct upon parenchymal injury. **a** Exemplified image of immunostained liver sections used for the analysis. Bile canaliculi (CEACAM1, red), nuclei of hepatocytes (HNF4α, blue), and bile ducts (CK19, green) were visualized. Scale bar, 100 μm. **b** Magnified images showing typical types of hepatocytes in different categories. White dotted lines delineate the minimum distance from the center of hepatocyte nuclei to bile canalicular networks or to bile ducts. White arrow points to a hepatocyte detached from both bile canaliculi and bile ducts (total bile networks). Scale bars, 20 μm. **c** Classification of hepatocytes by quadrants (Q1–Q4) according to the distances from the bile-excreting network structures. **d** Scatter plot results showing the hepatocyte status under the normal and liver injury conditions. The horizontal and vertical axes indicate minimum distances from the nucleus of hepatocytes to the edge of bile canalicular networks and that of BECs, respectively. Blue lines indicate the 20-μm thresholds. Quantification was done at a single-cell resolution, and each dot corresponds to a single hepatocyte. For each of the conditions, the data were acquired from *n* = 4 mice with five randomly chosen areas in liver sections analyzed per mouse, and all of those hepatocytes analyzed were shown en bloc in the plots (*n* = 18821, 14855, 8735, 22529, and 12293 hepatocytes for Normal, DDC 1 wk, DDC 8 weeks, TAA 1 week, and TAA 8 weeks, respectively). **e** Transition of the hepatocyte status during the course of liver injury. Mean data ± standard deviation of *n* = 4 mice are shown (*P = 0.0304 vs. 0 week; # *P* = 0.0304 vs. 1 week). **f** Evaluation for the significance of the correlation between the detachment from bile canalicular networks and the formation of connection with bile ducts. The data of hepatocytes detached from total bile networks were analyzed. The left columns correspond to the same experimental data as shown in the right graph of (**e**). The right columns show assumed data that was expected if the two distances were independent.

To strengthen the above observations, spatial information on the relationship between hepatocytes and the bile-excreting networks (i.e., biliary epithelia and bile canaliculi) was quantitatively analyzed on 2D immunofluorescence images (Fig. 4a) based on the following two features: the minimum distance between a hepatocyte (the center of a HNF4A^+^ nucleus) and bile canaliculi (CEACAM1^+^ structure), and the minimum distance between a hepatocyte and biliary tree (CK19^+^ structure) (Fig. 4b). We postulated the threshold distance for hepatocyte attachment to each structure to be 20 μm, as most of the hepatocytes (>99.9%) were located within this distance from bile canaliculi in the normal condition (Fig. 4d, leftmost graph). Hepatocytes were thus classified into four classes based on their attachment pattern, and the proportion of each class was determined (Fig. 4c, d). Of note, the proportion of hepatocytes that directly connected with bile ducts in normal conditions appeared very low albeit existing (0.063% on average; Fig. 4e, center graph); this should likely reflect the fact that only a limited fraction of the peri-portal hepatocyte population, among a large quantity of total hepatocytes, directly connect with the biliary system through a hemiductular structure known as the canal of Hering.

At the early phase (1 week) post injury induced by DDC or TAA, hepatocytes lost connection from bile canalicular networks (Fig. 4e, left graph). The hepatocytes that lost attachment to both bile canaliculi and the biliary tree increased at the early phase, but decreased later at 8 weeks of injury (Fig. 4e, right graph). At the late phase of injury, many hepatocytes established a de novo connection with a biliary tree (Fig. 4d, e, center), suggesting that BECs expanded to the collapsed area of bile canaliculi re-established the connectivity between hepatocytes and bile-excreting networks. Thus, the entire process can be summarized as follows: (1) virtually all hepatocytes are connected with bile canalicular networks in the non-injured condition; (2) in the early phase of liver injury, a substantial proportion of hepatocytes lose connection to the bile canalicular networks and hence being isolated form bile-excreting networks; (3) those hepatocytes that were isolated from bile-excreting networks decrease later owing to increased connection with the network of bile ducts. Importantly, such a process could be seen consistently in both TAA and DDC models. The correlation between the failure of bile canalicular networks and the formation of the biliary tree was further substantiated in these models by a Chi-square test for independence to evaluate the significance of their association (*p* = 0.005725 and 0.00928 for TAA and DDC models, respectively) (Fig. 4f).

### Destruction of bile canaliculi causes ductular reaction

**Fig. 5:**
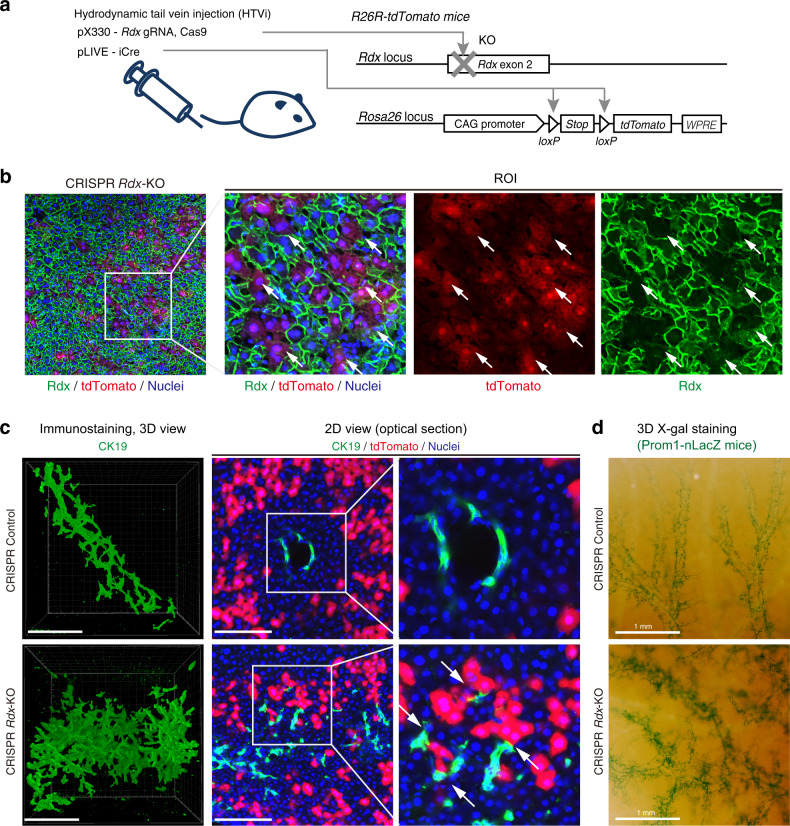
Destruction of bile canaliculi by *Rdx* deletion *in vivo* in mouse hepatocytes induces the ductular reaction. **a** *Rdx* knockout and cell-labeling strategies. Hydrodynamic tail vein injection (HTVi) was employed to deliver plasmids into mouse hepatocytes in vivo. Using R26R-tdTomato reporter mice as recipients, gene knockout and permanent cell labeling were induced simultaneously. **b** Validation of *Rdx* knockout in the mouse liver. At 2 weeks after the gene delivery, liver sections were prepared and expression of Rdx proteins (green) was analyzed by immunostaining, together with the tdTomato fluorescent signals (red) and nuclear staining by Hoechst3342 (blue). Representative image of *n* = 5 mice is shown. A region of interest (ROI) indicated by a white box in the left panel is magnified in the right panels. White arrows indicate tdTomato^+^ hepatocytes in which the Rdx expression was lost. Note that the loss of Rdx is observed solely in tdTomato^+^ cells. **c** Representative 3D images of the biliary tree in the liver of negative control mice (upper panels; *n* = 5 mice) and *Rdx*-knockout mice (lower panels; *n* = 5 mice). The left panels are 3D images of the biliary epithelial tissue (CK19 immunostaining), which were generated with IMARIS software (blend mode). Central panels show optical 2D sections, and magnified view thereof, of mouse livers. BECs are shown in green and gene-modified hepatocytes (tdTomato^+^ cells) in red. White arrows indicate the extended biliary branches that were located adjacent to the gene-modified hepatocytes. Scale bar, 100 μm. **d** Representative images of the biliary tree visualized by whole-mount X-gal staining in Prom1-CreERT2-nLacZ mice (*n* = 5 mice for each conditions). Scale bars, 1 mm.

Finally, to prove a causal relationship between the ductular reaction and impaired bile-excreting networks, we sought to clarify whether the destruction of the bile canalicular network alone is sufficient to induce the ductular reaction, as drug-induced injury should have affected hepatocytes in various other aspects. To examine the direct effect of canalicular collapse, we targeted a cytoskeletal protein, radixin (Rdx); loss of the corresponding gene in hepatocytes has been shown to impair bile canaliculi both structurally and functionally^31,32^. A CRISPR-Cas9 plasmid targeting *Rdx* was delivered into adult mouse hepatocytes in vivo via hydrodynamic tail vein injection (HTVi)^33,34^, in conjunction with a Cre-loxP–dependent cell-labeling system to monitor transduced hepatocytes using the R26R-tdTomato reporter mice as recipients (Fig. 5a). Due to the inherent nature of the HTVi-mediated in vivo delivery method, gene transduction in the liver parenchyma does not occur uniformly but in a mosaic pattern (Fig. 5c, red signals in central panels) and tends to be enriched around the CV but is less efficient around the PV. This feature of HTVi is suited for this experiment because it mimics the nature of destruction of bile canaliculi in the TAA model, in which the destruction occurs locally and specifically in peri-CV hepatocytes (Fig. 3a, b, and Supplementary Fig. 2).

Genomic DNA sequencing analyses on gene-transduced hepatocytes that were isolated based on tdTomato expression confirmed that multiple inserts/deletions occurred in the targeted *Rdx* gene (Supplementary Fig. 8a), while no substantial off-target effects were detected (Supplementary Table 1). Immunostaining of liver tissue sections confirmed that Rdx expression was diminished at the protein level in the transduced hepatocytes (Fig. 5b), which was observed even in bi-nucleated hepatocytes (Supplementary Fig. 8b, c). Importantly, the targeting of Rdx in hepatocytes did not cause any symptoms of hepatocyte injury or cholestasis (Supplementary Fig. 9).

At 4 weeks after HTVi, no apparent changes in the biliary tree were observed in the livers from the negative control group (Fig. 5c, upper panels). In stark contrast, drastic expansion of biliary tree structure was induced in *Rdx* knockout livers (Fig. 5c, lower panels). This phenotype was further confirmed at a different scale with the biliary tree being macroscopically visualized using a 3D imaging method based on whole-mount X-gal staining^30^ (Fig. 5d). These results clearly established a causal relationship between the collapse of the bile canalicular network and the ductular reaction. Intriguingly, the defect of bile canaliculi was induced in only a small fraction of hepatocytes in this experiment, but was sufficient to strongly induce a ductular reaction. It is also important to note that the branches of the biliary tree expanded so that they located adjacent to the gene-modified hepatocytes (Fig. 5c, lower panel, white arrows). This directional biliary remodeling fits well with the results of other experiments and strongly supports our hypothesis that expansion of the biliary tree is induced toward the collapsed area of bile canaliculi to restructure a complementary bile-excreting network in injured liver parenchyma (Supplementary Fig. 10).

## Discussion

It has recently come to our attention that the biliary epithelial tissue in the liver takes a much more complex and dynamic structure, rather than a simple and stable tube as depicted in many literatures, so that study of the tissue at the 3D level is becoming increasingly important^22,30,35–37^. Besides, it is difficult to trace the flow of bile, which is the primary role of the biliary tract, by using conventional histological methods. In this study, we developed and utilized multidimensional imaging methods for intravital imaging of the mouse liver and 3D immunofluorescence staining, which eventually revealed the role of the ductular reaction in the reconstruction and restoration of the functional biliary channel structure in the injured liver parenchyma.

The causal relationship between bile canalicular collapse and BEC expansion in the ductular reaction successfully explains the complex morphology of the biliary tree and its structural diversity in various injury models. That is, the impaired bile canalicular network may function as a “mold” that dictates the 3D architecture in the regeneration (or “casting”) of a functional bile duct. The structural pattern of bile canalicular destruction is unique to each type of liver injury, thus generating diverse “molds”.

Interestingly, intrahepatic bile ducts in teleosts, such as zebrafish, have a complex lattice-like network structure even under physiological conditions^38^, which resembles the biliary epithelial network formed upon the ductular reaction in the mouse liver. Whereas a bile canaliculus in mammals consists of the apical membranes of two adjacent hepatocytes, in zebrafish, it is formed by a single hepatocyte as a membrane invagination and does not construct network structure. Instead, the ends of the biliary epithelial network are contiguous with all hepatocytes and transport bile from discrete canaliculi. Phylogenetic analysis suggested that the teleost-type liver tissue architecture arose from the mammalian-type one during the course of evolution^39^. Although the exact factors that affected and favored such evolution remain unclear, these notions strongly support the idea that the biliary epithelial tissue remodeling is an adaptive response that is advantageous to counter some environmental stress condition.

In conclusion, our present study has revealed an unprecedented role of the ductular reaction in the reconstruction of the biliary tree structure to re-establish the functional bile-excreting networks in the injured liver. This provides a potential unifying explanation for induction of the ductular reaction in a wide range of liver diseases, where the existence of local and cryptic failure of bile excretion and transportation is key to provoke the adaptive tissue remodeling process. Remarkably, a variety of inherited and acquired liver disorders, including viral hepatitis, have been documented to affect hepatocyte polarity, thereby leading to canalicular disorganization^40^. This interpretation of the ductular reaction provides a fundamental insight into the epithelial tissue dynamics that underlie organ homeostasis and regeneration, and will lead to future development of novel diagnostic and therapeutic strategies for human liver diseases.

## Methods

### Animal models

Animal experiments were conducted in accordance with the Guideline for the Care and Use of Laboratory Animals of The University of Tokyo, under the approval of the Institutional Animal Care and Use Committee of Institute for Quantitative Biosciences (formerly Institute of Molecular and Cellular Biosciences), The University of Tokyo (approval numbers 2501, 2501–1, 2609, 2706, 2804, 2904, 3004 and 3004–1). R26R-tdTomato mice^41^ and Prom1-CreERT2-nLacZ mice^42^ were purchased from Jackson Laboratory (Bar Harbor, ME, U.S.A.). CK19-CreERT mice^43^ were a gift from Dr. Guoqiang Gu (Vanderbilt University Medical School, TN, U.S.A.). Wild-type C57BL/6J mice were purchased from CLEA Japan (Tokyo, Japan) and used at 8–10 weeks of age. Both males and females were used. Mice were fed a 0.1% DDC-containing diet (F-4643; Bio-Serv, Flemington, NJ, U.S.A.) to establish the DDC model. Mice were administered TAA (204–00881; Wako, Osaka, Japan; 300 mg/L) as drinking water to establish the TAA model. The duration of each injury model is indicated in each figure. CK19-CreERT;R26R-tdTomato mice were used to visualize BECs. Tamoxifen (T5648; Sigma, St. Louis, MO, U.S.A.) was dissolved into corn oil and administered via oral gavage (10 mg/20 g body weight). For serum biochemical analyses, blood was collected from mice under isoflurane inhalation anesthesia, and serum samples were prepared by centrifugal separation. Serum samples were analyzed by Oriental Yeast Co., Ltd. (Tokyo, Japan).

### Intravital imaging

Intravital imaging of the liver in live mice was performed using upright two-photon excitation laser scanning microscopy. In order to settle the mouse liver under an objective lens in an upright configuration, an organ-holding device was originally designed and handcrafted using inexpensive and easily accessible materials, including acrylic plates and a glass coverslip. Mice were anesthetized by isoflurane gas, and an incision was made in the abdomen to surgically expose the liver. The mouse was placed sideways, and the exposed liver was laid on an acrylic plate stage of the organ-holding device. The liver was overlaid with an observation window made of glass coverslip, and then subjected to two-photon microscopy. This intravital imaging platform could generally achieve observation at the depth of around 200 μm in a routine and reproducible manner, and up to around 500 μm at best, depending on experimental conditions such as fluorescence dyes used. The details of the construction of the organ-holding device and the procedure for attachment of the liver to the device will be described elsewhere (manuscript in preparation).

For two-photon microscopy, an FVMPE-RS or FV1000MPE instrument (Olympus, Tokyo, Japan) was used. CLF (451041; Corning, NY, U.S.A.) and CMFDA (21879; Sigma) were used to visualize bile excretion. Texas red-conjugated dextran (D1863; Invitrogen, Carlsbad, CA, U.S.A.) was used to visualize blood flow. Reagents were diluted in PBS and injected intravenously just before intravital imaging.

### 2D histological analyses using tissue sections

Either a pre-fixation or a post-fixation protocol was used according to antibody compatibility (Supplementary Table 2). For pre-fixation, mouse liver was perfused with 4% paraformaldehyde (PFA, 162–16065; Wako) via the PV and fixed with 4% PFA at 4 °C for 12 h. Fixed samples were immersed in 20% sucrose in PBS at 4 °C for 24 h. The samples were embedded in Tissue-Tek O.C.T. compound (Sakura Finetek Japan, Tokyo, Japan), then frozen. In the post-fixation protocol, mouse liver was immediately subjected to embedding and freezing in the O.C.T. compound. After sectioning, tissue slices were fixed with 4% PFA at room temperature for 5 min. Both pre-fixed and post-fixed samples were further treated in the same manner. Samples were incubated with blocking/permeabilization reagent (3% FBS, 0.2% Triton X-100, and 0.02% sodium azide in PBS) at room temperature for 5 min. The samples were incubated with primary antibody in blocking/permeabilization reagent for 12 h at 4 °C. After washing with PBS, the samples were incubated with the secondary antibody conjugated with Alexa Fluor 488, 555, or 647 (Thermo Fisher, Waltham, MA, U.S.A.), and Hoechst33342 (H1399; Thermo Fisher). To visualize the cell border of hepatocytes, actin filaments were labeled with Alexa Fluor 647-conjugated phalloidin (A22287, Thermo Fisher). Then, the samples were sealed with Fluoromount (Diagnostic BioSystems, Pleasanton, CA, U.S.A.).

For histological analyses shown in Supplementary Figs. 1–3, paraffin-embedded tissue sections were used. Liver tissue samples were pre-fixed with 4% paraformaldehyde, dehydrated, and then embedded in paraffin blocks. Consecutive tissue sections in 2-μm-thickness and 8-μm-thickness were prepared from each of the samples using a microtome. The 2-μm sections were subjected to hematoxylin and eosin staining using Mayer’s hemalum solution (109249, Merck) and 1% Eosin Y solution (Muto Pure Chemicals, Co. Ltd., Tokyo, Japan). The 8-μm sections were used for immunofluorescent staining after antigen retrieval by autoclaving (110 °C, 10 min) in TE buffer (pH 9.0), with primary antibodies listed in Supplementary Table 2.

### 3D immunohistology

3D immunohistological analysis was performed according to our original protocol^30^. Pre-fixed, frozen liver samples were prepared by the same protocol as that used for 2D immunohistology. Samples were cut into 200-μm-thick sections using a cryostat microtome. After washing with PBS, the samples were permeabilized with blocking/permeabilization reagent and subjected to primary antibody staining, washing, and secondary antibody staining. Nuclei were counterstained with Hoechst33342 or SYTOX Green (Thermo Fisher). All procedures were performed at 4 °C on a rocking device, and antibody concentrations were the same as those used for 2D immunohistology (Supplementary Table 2). After staining, samples were treated with SeeDB^44^ overnight. Images were acquired under a confocal microscope (FV-1000 or FV3000; Olympus) with a 30× silicone immersion lens (UPLSAPO30XS; Olympus). 3D images were reconstructed with IMARIS software (Bitplane, Zurich, Switzerland).

### Plasmid construction and in vivo gene delivery

CRISPR-Cas9 plasmids were constructed with pX330-U6-Chimeric_BB-CBh-hSpCas9, a backbone plasmid for the CRISPR-Cas9 system generated by and gifted from Dr. Feng Zhang^45^ (# 42230; Addgene). CRISPR DESIGN (http://crispr.mit.edu) was used for sgRNA design and off-target site prediction (Supplementary Table 1). To construct the *Rdx*-targeting plasmid, the following synthetic oligonucleotides were annealed and cloned into the BbsI sites of pX330: 5′-CACCGGCCATCCAGCCCAATACAAC-3′ and 5′-AAACGTTGTATTGGGCTGGATGGCC-3′. For the labeling of gene-delivered cells, pLIVE-iCre plasmid was injected with CRISPR-Cas9 construct into R26R-tdTomato mice. The *iCre* gene was amplified from pDIRE (# 26745; Addgene, gifted by Dr. Rolf Zeller)^46^ and cloned into the pLIVE vector backbone (Mirus Bio, Madison, WI, U.S.A.) to generate pLIVE-iCre plasmid. pX330-sgRdx and pLIVE-iCre were mixed and dissolved in TransIT-EE Hydrodynamic delivery solution (Mirus Bio) at 20 μg/ml and 5 μg/ml, respectively, and delivered to mouse hepatocytes in vivo by HTVi (2 ml plasmid solution/20 g body weight)^33,34^. pX330-sgGFP plasmid^33^ was used as a control.

### Quantification of imaging data

2D and 3D imaging data were quantified with Volocity (PerkinElmer, Waltham, MA, U.S.A.) and IMARIS, respectively. Data analysis, visualization, and statistical analyses were carried out with python (3.5) and its libraries; numpy (1.11.3), pandas (0.19.2), scipy (0.18.1), matplotlib (2.0.0), seaborne (0.7.1), and jupiter notebook.

### Statistics and reproducibility

All experiments were independently repeated using at least three mice per experimental groups. The exact numbers of mice and biological replicates used for each of the experiments are indicated in legends to the figures. Data are expressed as the mean ± standard deviation. The Mann–Whitney *U*-test (two-tailed) was used to evaluate the significance of the differences, and the differences were considered statistically significant at *P* < 0.05.

### Reporting summary

Further information on research design is available in the Nature Research Reporting Summary linked to this article.

## Supplementary information

Supplementary Information file

Reporting Summary

## Data Availability

The data that support the findings of this study are available from the corresponding author upon reasonable request. All data will be deposited at the institutional data repository at the University of Tokyo and will be publicly accessible following an embargo period.
