# Supplementary material for: Multidimensional imaging of liver injury repair in mice reveals fundamental role of the ductular reaction
Source: Commun Biol. 2020 Jun 5;3:289. doi: 10.1038/s42003-020-1006-1 (PMC7275065; doi:10.1038/s42003-020-1006-1)
Supplement: Supplementary file 1 — Supplementary Information file [file 42003_2020_1006_MOESM1_ESM.pdf]

Supplementary Information for

# Multidimensional imaging of liver injury repair in mice reveals fundamental role of the ductular reaction

**This PDF file includes:**

Supplementary Figs. 1 to 10

Supplementary Tables 1 and 2

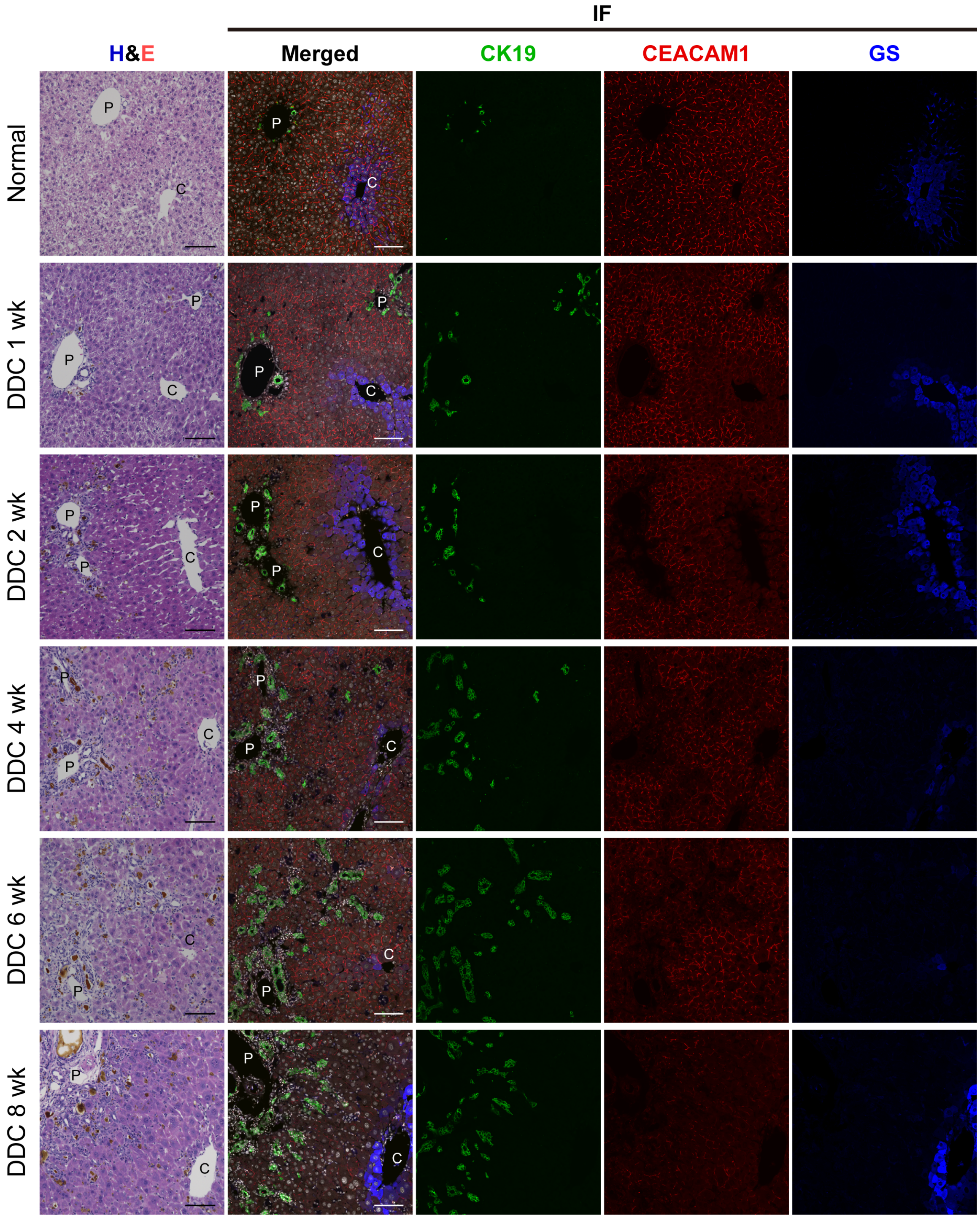
IF
H&E
Merged
CK19
CEACAM1
GS
Normal
DDC 1 wk
DDC 2 wk
DDC 4 wk
DDC 6 wk
DDC 8 wk
P
C

**Supplementary Fig. 1. | Histopathological progression in the DDC-induced cholestatic liver injury model in mice.** Mice were fed a control diet (Normal) or the 3,5-diethoxycarbonyl-1,4-dihydrocollidine (DDC)-containing diet for 1–8 weeks. Liver samples were collected at the indicated time points, and tissue sections were prepared and subjected to hematoxylin and eosin staining (H&E). Adjacent sections were used for immunofluorescent co-staining (IF) with the biliary epithelial marker CK19 (green), the bile canalicular marker CEACAM1 (red) and a marker for peri-central venous hepatocytes, GS (blue). Merged images for those three markers together with counterstaining for nuclei (Hoechst33342, white) are also indicated. Scale bars, 100 μm. P, portal vein; C, central vein. Shown are representative images for three or more mice analyzed per each time point.

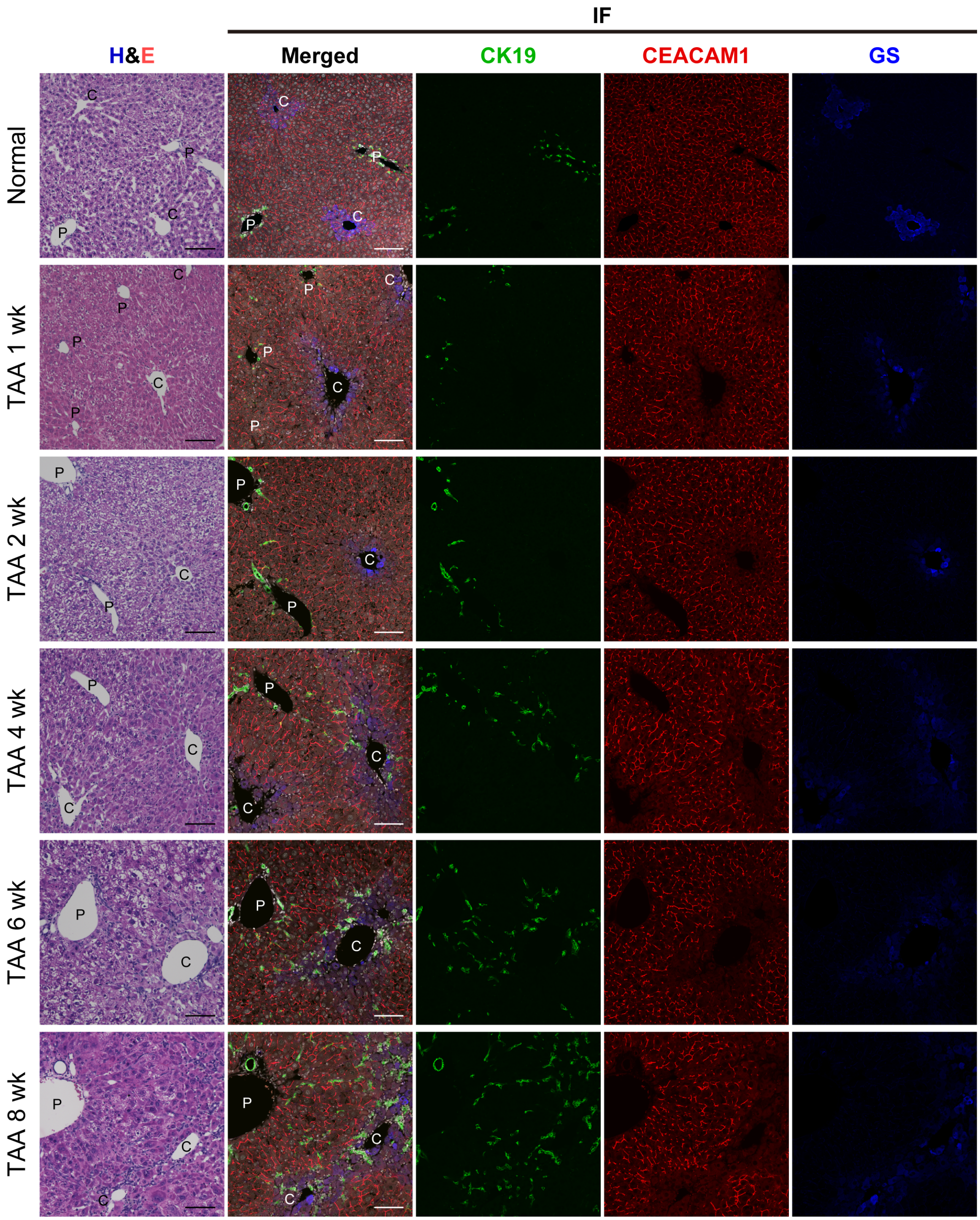
IF
H&E
Merged
CK19
CEACAM1
GS
Normal
TAA 1 wk
TAA 2 wk
TAA 4 wk
TAA 6 wk
TAA 8 wk

**Supplementary Fig. 2. | Histopathological progression in the TAA-induced liver injury model in mice.** Mice were administered with thioacetamide (TAA) in drinking water for 1–8 weeks or kept with normal water (Normal). Liver samples were collected at the indicated time points, and tissue sections were prepared and subjected to hematoxylin and eosin staining (H&E). Adjacent sections were used for immunofluorescent co-staining (IF) with the biliary epithelial marker CK19 (green), the bile canalicular marker CEACAM1 (red) and a marker for peri-central venous hepatocytes, GS (blue). Merged images for those three markers together with counterstaining for nuclei (Hoechst33342, white) are also indicated. Scale bars, 100 μm. P, portal vein; C, central vein. Shown are representative images for three or more mice analyzed per each time point.

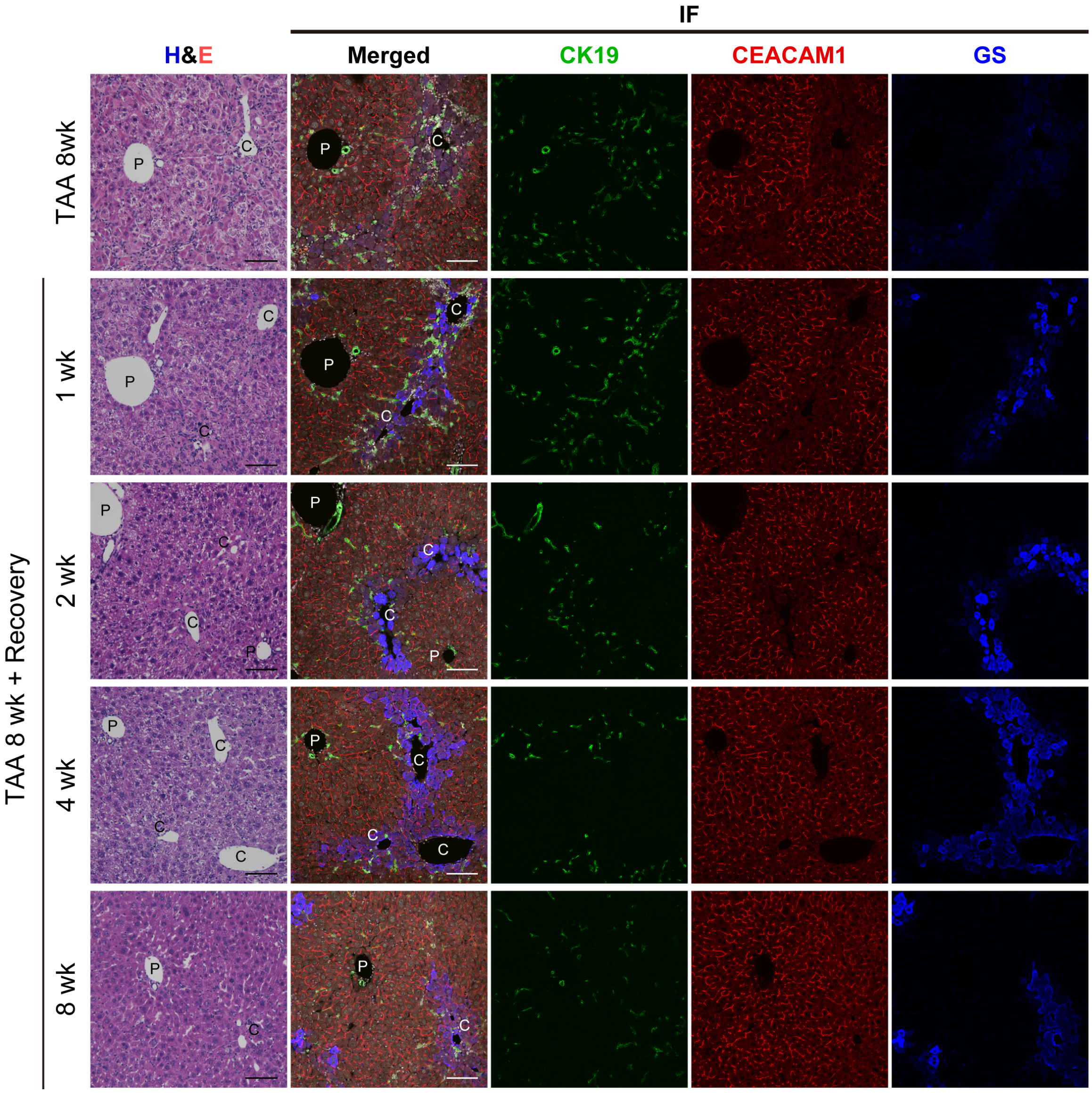

IF
H&E
Merged
CK19
CEACAM1
GS
TAA 8wk
TAA 8 wk + Recovery
1 wk
2 wk
4 wk
8 wk
P
C

**Supplementary Fig. 3. | Histopathological regression during the recovery phase from the TAA-induced chronic liver injury.** Mice were administered with TAA in drinking water for 8 weeks, and then switched to normal drinking water for recovery for 1–8 weeks. Liver samples were collected at the indicated time points, and tissue sections were prepared and subjected to hematoxylin and eosin staining (H&E). Adjacent sections were used for immunofluorescent co-staining (IF) with the biliary epithelial marker CK19 (green), the bile canalicular marker CEACAM1 (red) and a marker for peri-central venous hepatocytes, GS (blue). Merged images for those three markers together with counterstaining for nuclei (Hoechst33342, white) are also indicated. Scale bars, 100 μm. P, portal vein; C, central vein. Representative images for three or more mice analyzed per each time point are shown.

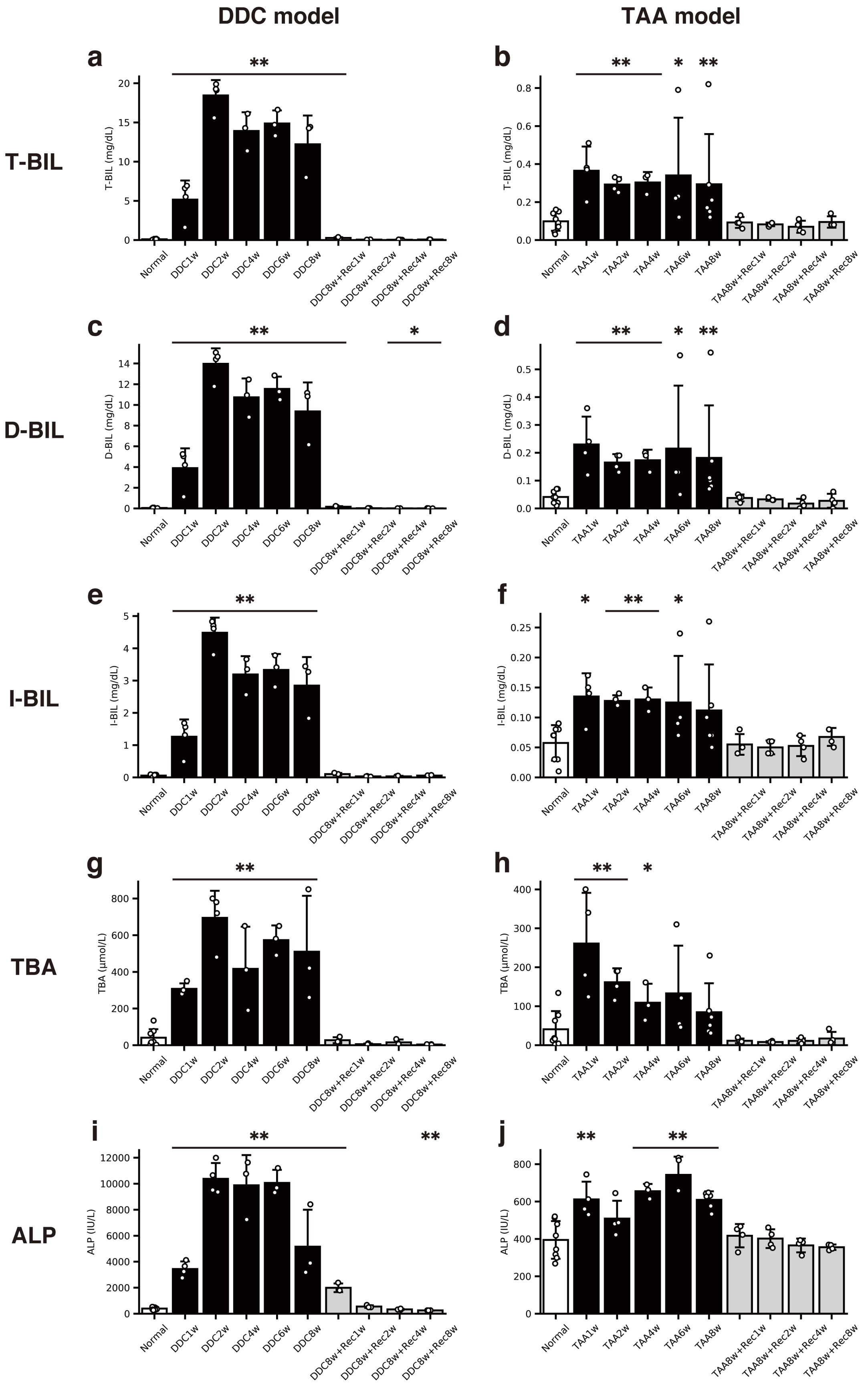
DDC model
TAA model
a
b
c
d
e
f
g
h
i
j
T-BIL
D-BIL
I-BIL
TBA
ALP
T-BIL (mg/dL)
D-BIL (mg/dL)
I-BIL (mg/dL)
TBA (μmol/L)
ALP (IU/L)
Normal
DDC1w
DDC2w
DDC4w
DDC6w
DDC8w
DDC8w+Rec1w
DDC8w+Rec2w
DDC8w+Rec4w
DDC8w+Rec8w
TAA1w
TAA2w
TAA4w
TAA6w
TAA8w
TAA8w+Rec1w
TAA8w+Rec2w
TAA8w+Rec4w
TAA8w+Rec8w

**Supplementary Fig. 4. | Distinct levels of cholestatic conditions in the DDC-induced and TAA-induced liver injury models.** Mice were subjected to the liver injury models induced by the DDC-containing diet (**a**, **c**, **e**, **g**, **i**) or the TAA-containing water (**b**, **d**, **f**, **h**, **j**) for up to 8 weeks (black bars), and then returned to recovery conditions (Rec) with a control diet and normal water for up to 8 weeks (gray bars). Blood samples were collected and the serum levels of total bilirubin (T-BIL; **a**, **b**), direct bilirubin (D-BIL; **c**, **d**), indirect bilirubin (I-BIL; **e**, **f**), total bile acid (TBA, **g**, **h**), and alkaline phosphatase (**i**, **j**) were analyzed. Data represent the mean ± standard deviation for n samples; Normal, n = 8; DDC 1w, n = 4; DDC 2w, n = 4; DDC 4w, n = 3; DDC 6w, n = 3; DDC 8w, n = 3; DDC 8w + Rec 1w, n = 3; DDC 8w + Rec 2w, n = 3; DDC 8w + Rec 4w, n = 3; DDC 8w + Rec 8w, n = 3; TAA 1w, n = 4; TAA 2w, n = 4; TAA 4w, n = 3; TAA 6w, n = 4; TAA 8w, n = 6; TAA 8w + Rec 1w, n = 4; TAA 8w + Rec 2w, n = 4; TAA 8w + Rec 4w, n = 4; and TAA 8w + Rec 8w, n = 4. Statistical significance was evaluated by the Mann-Whitney U test (two-tailed). * $P < 0.05$ vs. Normal; ** $P < 0.01$ vs. Normal. Note that the Y-axis scales are different between the DDC and TAA models for each of the test items.

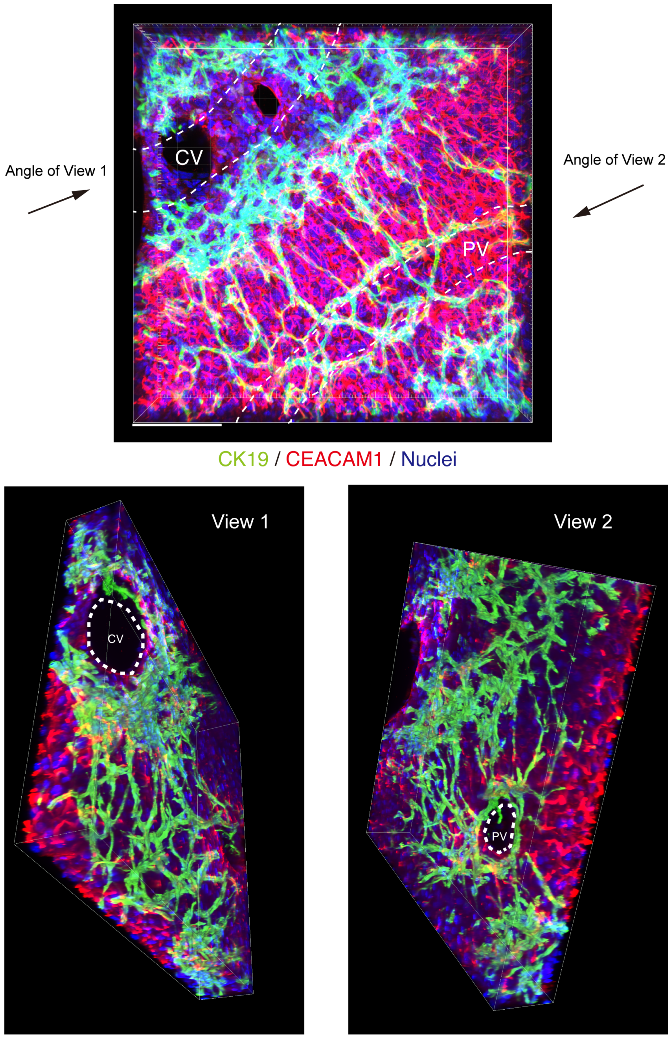


**Supplementary Fig. 5. | The biliary tree extends branches from the PV area toward the CV area and forms a network structure around the CV upon TAA-induced liver injury.** The same 3D visualization data as shown in Fig. 3a (upper panel) was rotated for observation from two different angles of view (View 1 and View 2; bottom panels) to indicate the positions of blood vessels. Liver tissue from TAA-injured mice (8 weeks) was co-immunostained for bile canaliculi (CEACAM1, red), biliary epithelial cells (CK19, green), and nuclei (Hoechst3342, blue). Positions of blood vessels of the portal vein (PV) and the central vein (CV) are highlighted in white dotted lines. Scale bars, 100 μm.

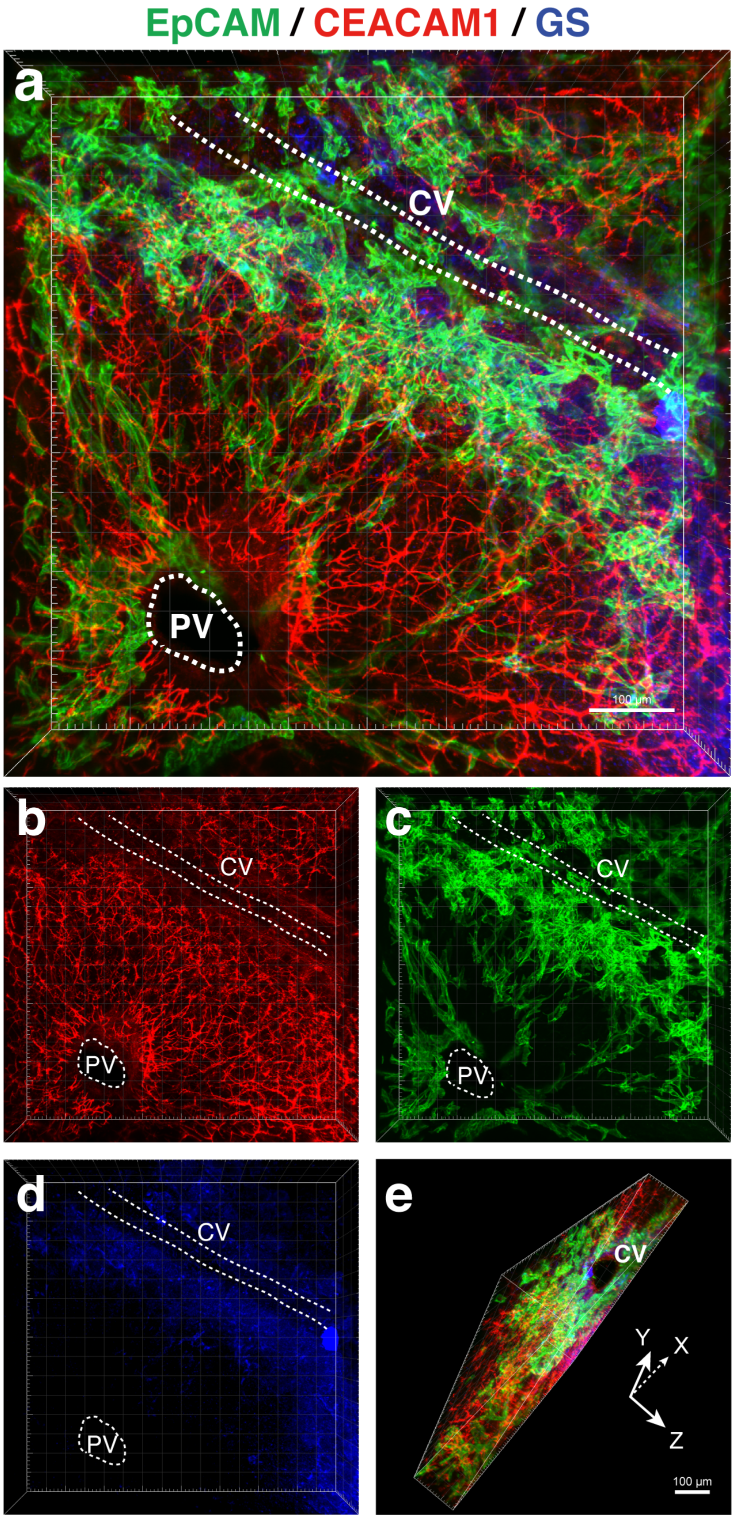

EpCAM / CEACAM1 / GS
a
CV
PV
100 µm
b
CV
PV
c
CV
PV
d
CV
PV
e
CV
Y
X
Z
100 µm

**Supplementary Fig. 6. | The biliary tree extends branches from the PV area toward the CV area and forms a network structure around the CV upon TAA-induced liver injury.** Liver tissue from TAA-injured mice (8 weeks) was co-immunostained with the bile canalicular marker CEACAM1 (**a**, **b**, **e**; red), the biliary epithelial marker EpCAM (**a**, **c**, **e**; green) and a marker for peri-central venous hepatocytes, GS (**a**, **d**, **e**; blue,). Panel **a** represents the merged image for single channel images shown in **b**–**d**. Panel **e** indicates the same 3D image as shown in panel **a**, observed from a different angle of view. Positions of blood vessels of the portal vein (PV) and the central vein (CV) are highlighted in white dotted lines. Scale bars, 100 μm. Note the presence of clear blood vessel lumens of the PV and CV in **a** and **e**, respectively. Expression of GS surrounding the CV can discriminate it from the PV.

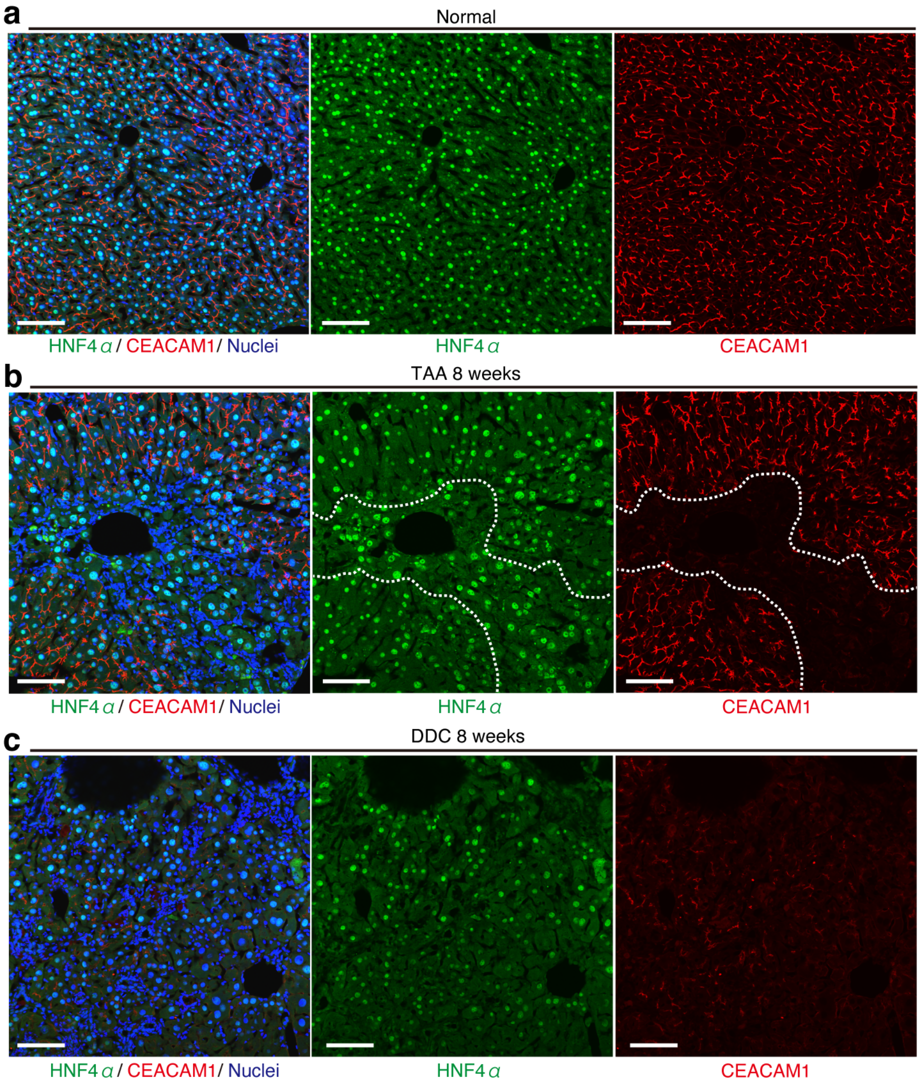


**Supplementary Fig. 7. | Collapse of bile canaliculi is not due to the loss of hepatocytes.** **a–c** 2D immunostaining images of bile canaliculi (CEACAM1, red), nuclei of hepatocytes (HNF4A, green), and nuclei (Hoechst33342, blue). **a** Mouse liver tissue in normal conditions (representative image for n = 4 mice analyzed). **b** Mouse liver tissue of TAA injury model at 8 weeks (n = 4 mice). White dotted lines delineate the area where bile canaliculi are collapsed. Note that hepatocytes do exist in those areas where the canalicular structure is nearly completely lost. **c** Mouse liver tissue of DDC injury model at 8 weeks (n = 4 mice). Bile canaliculi are collapsed overall. Scale bars, 100 μm.

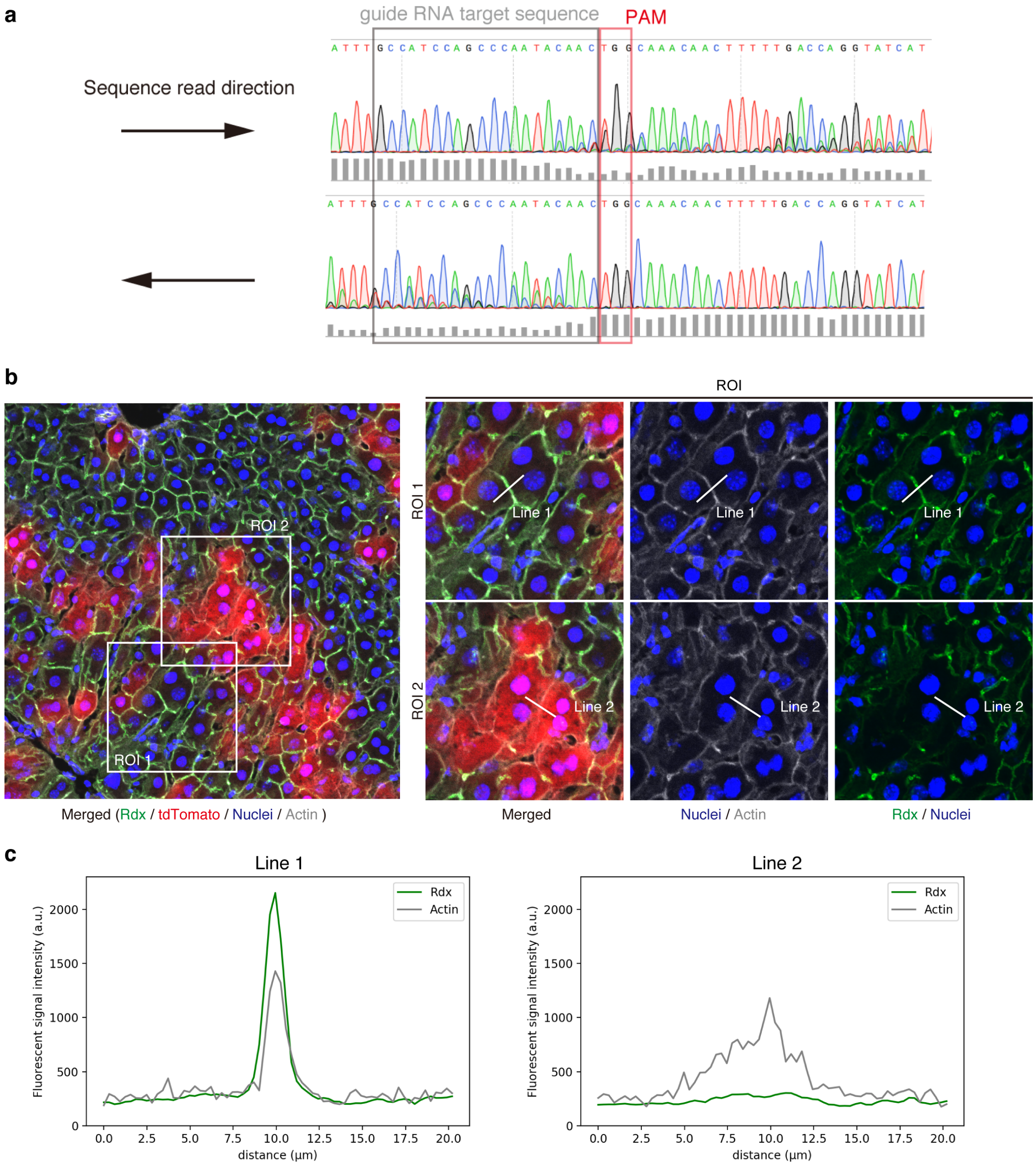
a
guide RNA target sequence
PAM
Sequence read direction
b
ROI
ROI 2
ROI 1
ROI 1
ROI 2
Line 1
Line 2
Merged (Rdx / tdTomato / Nuclei / Actin )
Merged
Nuclei / Actin
Rdx / Nuclei
c
Line 1
Line 2
Rdx
Actin
Fluorescent signal intensity (a.u.)
distance (μm)

**Supplementary Fig. 8. | Validation of *Rdx* knockout. a** CRISPR/Cas9-mediated genome editing in mouse hepatocytes was examined by DNA sequencing. Genomic DNA was purified from hepatocytes with hydrodynamic tail vein injection (HTVi)-mediated transduction of the *Rdx*-targeting construct (tdTomato$^+$ hepatocytes) isolated by FACS, and the *Rdx* gene was amplified and sequenced from both directions. Representative sequencing chromatogram and quality scores (gray bars) for n = 3 mice analyzed are shown. **b**, **c** *Rdx* knockout was achieved in bi-nucleated hepatocytes. **b** At 2 weeks after the HTVi-mediated transduction of the *Rdx*-targeting construct, liver samples were harvested and tissue sections were analyzed by immunofluorescent staining for Rdx proteins (green) together with the tdTomato fluorescence (red), actin (gray) and nuclei (Hoechst3342, blue). Regions of interest (ROI 1 and ROI 2) indicated by white boxes in the left panel are magnified in the right panels. **c** Fluorescent signal intensities for Rdx (green line graph) and actin (gray line graph) were quantitated along the white lines (Line 1 and Line 2) depicted in **b**. While the Rdx expression was generally observed to be localized coincidently with actin cytoskeleton at the border of normal hepatocytes (tdTomato$^-$; ROI 1 and Line 1), it could be substantially lost at the cellular border formed by two adjacent bi-nucleated hepatocytes with successful gene transduction (tdTomato$^+$; ROI 2 and Line 2). Representative data for n = 5 mice are shown.

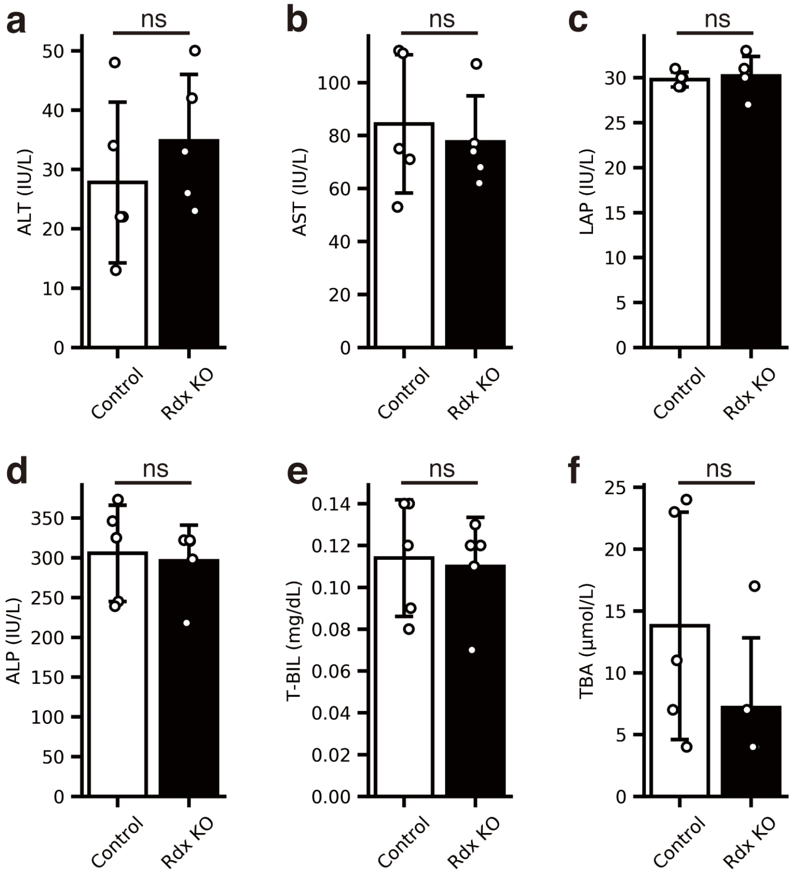


**Supplementary Fig. 9. | HTVi-mediated *Rdx* knockout in hepatocytes does not cause hepatotoxicity or cholestasis.** Serum samples were collected from mice at 2 weeks after the HTVi-mediated transduction of the *Gfp*-targeting construct (Control) or *Rdx*-targeting construct (Rdx KO), and subjected to biochemical analysis of markers for hepatocyte injury and cholestasis: **a**, alanine aminotransferase (ALT); **b**, aspartate aminotransferase (AST); **c**, leucine aminopeptidase (LAP); **d**, alkaline phosphatase (ALP); **e**, total bilirubin (T-BIL); and **f**, total bile acid (TBA). Data represent the mean ± standard deviation of n = 5 mice for each of the conditions. ns, not statistically significant by the Mann-Whitney U test (two-tailed; $P > 0.05$). The exact P-values were as follows: ALT, 0.2778; AST, 0.6905; LAP, 0.6667; ALP, 0.3810; T-BIL, 0.6587; and TBA, 0.2063.

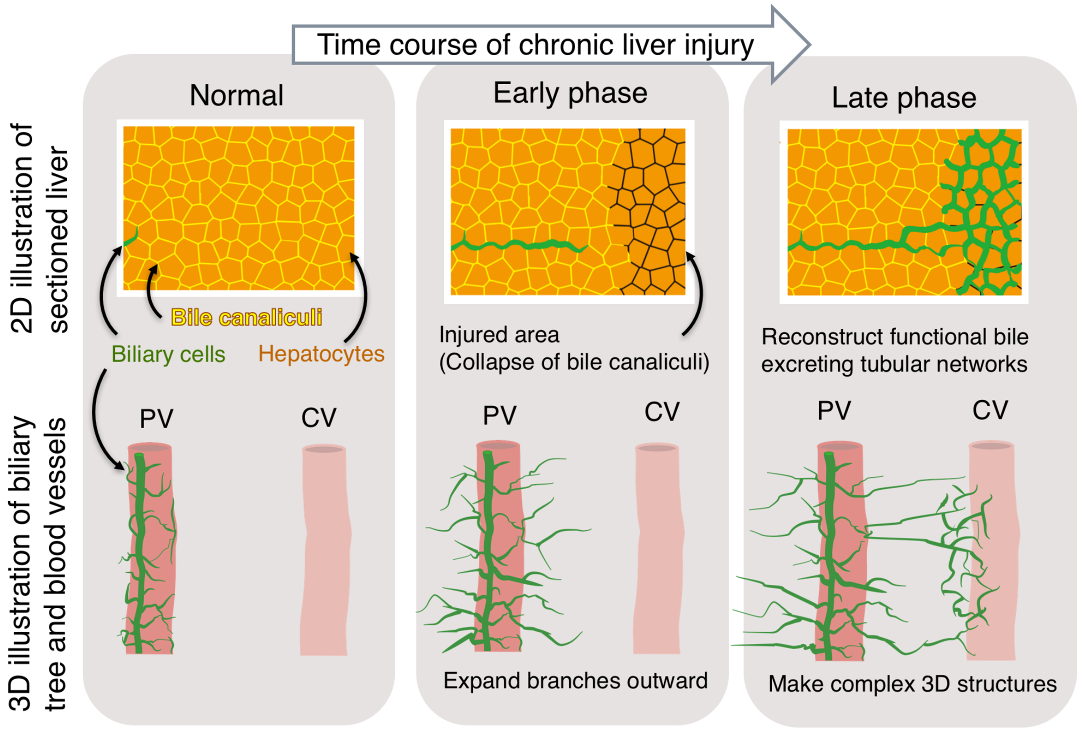


**Supplementary Fig. 10. | Schematic illustration of our hypothesis and conclusion.**

Under the non-injured condition, hepatocytes are connected with bile canalicular networks, which transport bile to bile ducts that consist of biliary epithelial cells and localize in the peri-portal region (left panel). In the early phase of liver injury, damaged hepatocytes suffer from canalicular collapse and lose connection to the bile canalicular networks, hence being isolated form the bile-excreting network. Bile ducts start to expand their branches toward the injured area with the collapse of bile canaliculi (center panel). Owing to such an ‘adaptive remodeling’ process of the biliary epithelium, those hepatocytes in the injured area acquire alternative connection with the biliary tree and thereby being re-united with the bile-excreting network system (right panel). PV, portal vein; CV, central vein.

**Supplementary Table 1. | Prediction and validation of off-target effects of CRISPR/Cas9-mediated *Rdx* gene knockout**

# of off target candidates, 99; 6 are in genes

| Rank | Sequence | Score | Mismatch | Gene | Locus (in mm9) | Primers<br>Forward | <br>Reverse | Amplicon size (nt) |
|---|---|---|---|---|---|---|---|---|
| On target | GCCATCCAGCCCAATACAAC(TGG) | 88 | - | NM_001104617 | chr9:+51871724 | TTGAACTCAGGTCATGAGGCTTTCA | CCACTACAGTAAGGAGTGGAAACA | 836 |
| Off targets | | | | | | | | |
| 1 | TGAATCCAGCCCAATACAAG(AAG) | 0.7 | 4MMs [1:2:3:20] | | chr12:+54169882 | TCTATCCCAGGGAACTGGTGAGC | GCTTCAACCTTGCTAAGTAGCTCTG | 898 |
| 2 | GGCATACAGCCCTATACAAC(TGG) | 0.7 | 3MMs [2:6:13] | | chr9:+51282024 | CTGGGCCCTCCTATGACCATTAG | GCTCTAACTGACAAGAGACAGAGGG | 920 |
| 3 | GCCAGCCAGCCCAATAAAAA(CAG) | 0.5 | 3MMs [5:17:20] | | chr3:+158352001 | CAGTCTCCATCCTTCCTACGGTAG | CCAGACACCTGGTTGTATAAGGAATG | 879 |
| 4 | ATCATACAGCTCAATACAAC(AGG) | 0.5 | 4MMs [1:2:6:11] | | chr4:+123053096 | ACCTGCTTGACTTTGGGAAAGGTGC | TGTACATGGGGACCTTGACCTCGTC | 905 |
| 5 | ACCATCATGCCCAATACAAT(GGG) | 0.5 | 4MMs [1:7:8:20] | | chr17:-13878817 | TCTCTGTTGTGCTTCTCCACAGAG | AACAGGCATTGAGTTGCAGAAGAC | 827 |
| (protein-coding) | | | | | | | | |
| 16 | GCCACCCTGTCCAATTCAAC(AGG) | 0.2 | 4MMs [5:8:10:16] | NR_037989 | chr1:-103065333 | TCCTTCATTGTCTCCACCTGGCG | TTAGTCAGGTGGTCCTGAACAGTG | 879 |
| 32 | GGCATCCAGCAGAATACAGC(TGG) | 0.1 | 4MMs [2:11:12:19] | NM_016675 | chrX:+136344579 | AGAATGACTTTGGCCATTGGATGG | AGGAAACCCTTGTCTAGGGCTCC | 826 |
| 41 | GCCATTCAGCCCAACACCAC(TGG) | 0.1 | 3MMs [6:15:18] | NM_010833 | chrX:+93337244 | TGGAACTGGCTGTTGTTAAAGGGTG | ATGTCACACACAGTCTGCACATGG | 919 |
| 42 | GCCACGCAGCCCAAGAGAAC(AGG) | 0.1 | 4MMs [5:6:15:17] | NM_008551 | chr1:+132951552 | CAGTTCAAGCAGGAGTCCCTGTAG | GCCTTGTCTGTCTGTCCCTCACTG | 836 |
| 59 | GCCATCCAGCCAAACACCAC(CGG) | 0.1 | 3MMs [12:15:18] | NM_009510 | chr17:-6963842 | CGGTACCCTGTATAGTTTCCACTTGAC | CGTGACCCTCCTGCTGGTACCTC | 866 |
| 90 | GCCATCCAGGCCAATCGCAC(TGG) | 0 | 4MMs [10:16:17:18] | NM_001077411 | chr3:+89008069 | GCCTGAGACATCTTTGGGCTGGG | GGGGCATAAGTTACAGTGTACCACC | 809 |

**Supplementary Table 2. | List of antibodies used in this study**

| Target | Company/Source | Catalog # | Host animal | Application* | Fixation | Dilution |
|---|---|---|---|---|---|---|
| CK19 | In-house ** | (n.a.) | Rabbit | 2D, 3D | Pre-fixation or Post-fixation | 1:2000 |
| CEACAM1 | R&D Systems | AF6480 | Rat | 2D, 3D | Pre-fixation or Post-fixation | 1:200 |
| EpCAM (CD326) | BD Biosciences (Pharmingen) | 552370 (clone G8.8) | Rat | 3D | Pre-fixation | 1:200 |
| GS | Merck (Chemicon) | MAB302 (clone GS-6) | Mouse | 2D | Pre-fixation | 1:500 |
| GS | Abcam | ab73593 | Rabbit | 3D | Pre-fixation | 1:200 |
| HNF4A | Santa Cruz | sc-6556 (C-19) | Goat | 2D | Post-fixation | 1:200 |
| HNF4A | Santa Cruz | sc-8987 (H-171) | Rabbit | 2D | Pre-fixation | 1:200 |
| Rdx | Abcam | ab52495 | Rabbit | 2D | Post-fixation | 1:200 |

* 2D, immunostaining for conventional (8–10 μm) tissue sections; 3D, immunostaining for 200-μm-thick sections

** Tanimizu, T., Nishikawa, M., Saito, H., Tsujimura, T. & Miyajima, A. Isolation of hepatoblasts based on the expression of Dlk/Pref-1. *J Cell Sci* **116**, 1775-1786 (2003).
