# Supplementary material for: Multidimensional imaging of liver injury repair in mice reveals fundamental role of the ductular reaction
Source: Commun Biol. 2020 Jun 5;3:289. doi: 10.1038/s42003-020-1006-1 (PMC7275065; doi:10.1038/s42003-020-1006-1)
Supplement: Supplementary file 2 — Reporting Summary [file 42003_2020_1006_MOESM2_ESM.pdf]

nature research

Corresponding author(s): Tohru Itoh

Last updated by author(s): Apr 30, 2020

Reporting Summary

Nature Research wishes to improve the reproducibility of the work that we publish. This form provides structure for consistency and transparency in reporting. For further information on Nature Research policies, see Authors & Referees and the Editorial Policy Checklist.

Statistics

For all statistical analyses, confirm that the following items are present in the figure legend, table legend, main text, or Methods section.

n/a	Confirmed	
☐	☒	The exact sample size (*n*) for each experimental group/condition, given as a discrete number and unit of measurement
☐	☒	A statement on whether measurements were taken from distinct samples or whether the same sample was measured repeatedly
☐	☒	The statistical test(s) used AND whether they are one- or two-sided<br>*Only common tests should be described solely by name; describe more complex techniques in the Methods section.*
☒	☐	A description of all covariates tested
☒	☐	A description of any assumptions or corrections, such as tests of normality and adjustment for multiple comparisons
☐	☒	A full description of the statistical parameters including central tendency (e.g. means) or other basic estimates (e.g. regression coefficient) AND variation (e.g. standard deviation) or associated estimates of uncertainty (e.g. confidence intervals)
☐	☒	For null hypothesis testing, the test statistic (e.g. *F*, *t*, *r*) with confidence intervals, effect sizes, degrees of freedom and *P* value noted<br>*Give P values as exact values whenever suitable.*
☒	☐	For Bayesian analysis, information on the choice of priors and Markov chain Monte Carlo settings
☒	☐	For hierarchical and complex designs, identification of the appropriate level for tests and full reporting of outcomes
☒	☐	Estimates of effect sizes (e.g. Cohen's *d*, Pearson's *r*), indicating how they were calculated

Our web collection on statistics for biologists contains articles on many of the points above.

Software and code

Policy information about availability of computer code

Data collection	2D and 3D imaging data were quantified with Volocity (PerkinElmer, Waltham, MA, U.S.A.) and IMARIS (Bitplane, Zurich, Switzerland), respectively. Data analysis, visualization, and statistical analyses were carried out with python (3.5) and its libraries; numpy (1.11.3), pandas (0.19.2), scipy (0.18.1), matplotlib (2.0.0), seaborne (0.7.1), and jupiter notebook.
Data analysis	2D and 3D imaging data were quantified with Volocity (PerkinElmer, Waltham, MA, U.S.A.) and IMARIS (Bitplane, Zurich, Switzerland), respectively. Data analysis, visualization, and statistical analyses were carried out with python (3.5) and its libraries; numpy (1.11.3), pandas (0.19.2), scipy (0.18.1), matplotlib (2.0.0), seaborne (0.7.1), and jupiter notebook.

For manuscripts utilizing custom algorithms or software that are central to the research but not yet described in published literature, software must be made available to editors/reviewers. We strongly encourage code deposition in a community repository (e.g. GitHub). See the Nature Research guidelines for submitting code & software for further information.

Data

Policy information about availability of data

All manuscripts must include a data availability statement. This statement should provide the following information, where applicable:

- Accession codes, unique identifiers, or web links for publicly available datasets
- A list of figures that have associated raw data
- A description of any restrictions on data availability

The data that support the findings of this study are available from the corresponding author upon reasonable request.

Field-specific reporting

Please select the one below that is the best fit for your research. If you are not sure, read the appropriate sections before making your selection.

☒ Life sciences ☐ Behavioural & social sciences ☐ Ecological, evolutionary & environmental sciences

For a reference copy of the document with all sections, see nature.com/documents/nr-reporting-summary-flat.pdf

Life sciences study design

All studies must disclose on these points even when the disclosure is negative.

Sample size	Sample sizes of sufficient statistical power were chosen based on previously published studies using similar analyses (PMIDs 23322300, 25572923, 27431614, 29404454, 29523685).
Data exclusions	No individual samples, animals or data were excluded from the analyses.
Replication	For each series of experiments, attempts at replication were successful. A statement describing experimental replication is included in the Methods section and the legend to each figure.
Randomization	Male and female mice were randomly chosen for inclusion in experiments and randomly assigned to each experimental/control group.
Blinding	Researchers were not blinded when analyzing results.

Reporting for specific materials, systems and methods

We require information from authors about some types of materials, experimental systems and methods used in many studies. Here, indicate whether each material, system or method listed is relevant to your study. If you are not sure if a list item applies to your research, read the appropriate section before selecting a response.

Materials & experimental systems

n/a	Involved in the study	
☐	☒	Antibodies
☒	☐	Eukaryotic cell lines
☒	☐	Palaeontology
☐	☒	Animals and other organisms
☒	☐	Human research participants
☒	☐	Clinical data

Methods

n/a	Involved in the study	
☒	☐	ChIP-seq
☒	☐	Flow cytometry
☒	☐	MRI-based neuroimaging

Antibodies

Antibodies used	We used the following primary antibodies for staining of tissue samples. They are listed as antigen first, followed by dilution, host, supplier, and catalog number as applicable:<br>CK19, 1:2000, Rabbit, In-house (Tanimizu N. et al., J Cell Sci, 116:1775, 2003).<br>CEACAM1, 1:200, Rat, R&D Systems, AF6480.<br>EpCAM, 1:200, Rat, BD Biosciences, 552370 (clone G8.8).<br>GS, 1:500, Mouse, Merck (Chemicon), MAB302 (clone GS-6).<br>GS, 1:200, Rabbit, Abcam, ab73593.<br>HNF4A, 1:200, Goat, Santa Cruz, sc-6556 (C-19).<br>HNF4A, 1:200, Rabbit, Santa Cruz, sc-8987 (H-171).<br>Rdx, 1:200, Rabbit, Abcam, ab52495.
Validation	All antibodies (except anti-CK19) are commercially available and validation is provided in the data sheets of the manufacturer. Anti-CK19 antibody was initially described in Tanimizu N. et al. (J Cell Sci, 116:1775, 2003, PMID 12665558) and has been validated in the following studies to specifically label biliary epithelial cells in the mouse liver (Tanaka M. et al., Mech Dev, 126:665, 2009, PMID 19527784; Okabe M. et al., Development, 136:1951, 2009, PMID 19429791) .

Animals and other organisms

Policy information about studies involving animals; ARRIVE guidelines recommended for reporting animal research

Laboratory animals	Animal experiments were conducted in accordance with the Guideline for the Care and Use of Laboratory Animals of The University of Tokyo, under the approval of the Institutional Animal Care and Use Committee of Institute for Quantitative Biosciences (formerly Institute of Molecular and Cellular Biosciences), The University of Tokyo (approval numbers 2501, 2501-1,

2609, 2706, 2804, 2904, 3004 and 3004-1). R26R-tdTomato mice (ref. 41) and Prom1-CreERT2-nLacZ mice (ref. 42) were purchased from Jackson Laboratory (Bar Harbor, ME, U.S.A.). CK19-CreERT mice 43 were a gift from Dr. Guoqiang Gu (Vanderbilt University Medical School, TN, U.S.A.). Wild-type C57BL/6J mice were purchased from CLEA Japan (Tokyo, Japan) and used at 8 to 10 weeks of age. Both males and females were used. Mice were fed a 0.1% DDC-containing diet (F-4643; Bio-Serv, Flemington, NJ, U.S.A.) to establish the DDC model. Mice were administered TAA (204-00881; Wako, Osaka, Japan; 300 mg/L) as drinking water to establish the TAA model. The duration of each injury model is indicated in each figure. CK19-CreERT;R26R-tdTomato mice were used to visualize BECs. Tamoxifen (T5648; Sigma, St. Louis, MO, U.S.A.) was dissolved into corn oil and administered via oral gavage (10 mg/20 g body weight). For serum biochemical analyses, blood was collected from mice under isoflurane inhalation anesthesia, and serum samples were prepared by centrifugal separation. Serum samples were analyzed by Oriental Yeast Co., Ltd. (Tokyo, Japan).

Wild animals	The study did not involve wild animals.
Field-collected samples	The study did not involve samples collected from the field.
Ethics oversight	Institute for Quantitative Biosciences (formerly Institute of Molecular and Cellular Biosciences), The University of Tokyo

Note that full information on the approval of the study protocol must also be provided in the manuscript.
